# Evolving Principles for Oral Squamous Cell Carcinoma Screening Programs

**DOI:** 10.3390/cancers18091462

**Published:** 2026-05-02

**Authors:** Alan Roger Santos-Silva, Joel B. Epstein, Luiz P. Kowalski, Thaís Cristina Esteves-Pereira, Ana Carolina Prado-Ribeiro, Manoela Domingues Martins, Marcio Ajudarte Lopes, Thomas P. Sollecito

**Affiliations:** 1 Oral Diagnosis Department, Piracicaba Dental School, University of Campinas, Piracicaba, SP 13414-903, Brazilmalopes@fop.unicamp.br (M.A.L.); 2 National Institute of Science and Technology in Translational Biophotonics in Dentistry (INCT-BIOFOTO BUCAL), Porto Alegre, RS 90035-003, Brazil; manoela.martins@ufrgs.br; 3 City of Hope National Medical Center, Duarte, CA 91010, USA; jepstein@coh.org; 4 Cedars-Sinai Health System, Los Angeles, CA 90048, USA; 5 Department of Head and Neck Surgery, Sao Paulo University Medical School, São Paulo, SP 01246-903, Brazil; lp_kowalski@uol.com.br; 6 Dental Oncology Service, Instituto do Câncer do Estado de São Paulo, Faculdade de Medicina da Universidade de São Paulo (ICESP-FMUSP), São Paulo, SP 01246-000, Brazil; 7 Oral Pathology Department, School of Dentistry, Federal University of Rio Grande Do Sul, Porto Alegre, RS 90035-003, Brazil; 8 Department of Oral Medicine, University of Pennsylvania School of Dental Medicine, Philadelphia, PA 19104-6030, USA; tps@upenn.edu

**Keywords:** mouth neoplasms, early detection of cancer, mass screening, health services accessibility

## Abstract

**Purpose**: Oral squamous cell carcinoma (OSCC) carries a substantial burden in low- and middle-income countries as well as underserved subpopulations within high-income settings, where structural barriers contribute to worse outcomes. While evidence supports targeted screening of high-risk groups, practical guidance for designing organized, quality-assured programs remains limited. This review proposes a framework to translate contemporary cancer-screening principles into operational criteria for OSCC. **Methods**: A review following the Scale for the Assessment of Narrative Review Articles was conducted. Conceptual papers, international evaluations, implementation studies, and programmatic guidance were included. The evidence was synthesized narratively, with emphasis on contemporary cancer-screening principles, implementation frameworks, and their applicability to OSCC. **Results**: Clinical oral examination can improve the detection of OSCC in early stages and reduce mortality among high-risk groups when embedded in coordinated care pathways. Effective programs require governance structures, screening policies, risk-stratified approaches, and robust information systems capable of call-recall, referral tracking, and quality monitoring. Dental schools and academic clinics might play a role as regional hubs and represent feasible anchors for programs within mixed health systems. **Conclusions**: Aligning core OSCC-screening principles with operational enablers offers a practical pathway to develop context-appropriate programs that strengthen capacity, promote equity, and generate evidence for responsible scale-up.

## Simple Summary

Oral squamous cell carcinoma (OSCC) is the most common malignancy of the oral cavity, with a disproportionate burden in low- and middle-income countries and among underserved populations in high-income settings. Tobacco and alcohol consumption are historically the primary risk factors, and primary prevention focuses on their reduction and cessation. Early detection through screening, particularly among high-risk individuals using clinical oral examination, has the potential to reduce mortality. This narrative review provides a concise overview of the current evidence on oral cancer screening and proposes a practical framework for implementing OSCC-organized screening programs in resource-constrained settings. The proposed framework outlines key domains including target populations, screening test performance, infrastructure, ethics, economic considerations, and quality assurance, with a focus on feasibility in resource-constrained settings.

## 1. Introduction

Oral squamous cell carcinoma (OSCC) remains a major global public health challenge, especially in South Asia and Latin America, with disproportionate incidence and mortality in vulnerable populations, particularly in populations with high exposure to tobacco and alcohol and those experiencing socioeconomic disadvantage [1,2,3]. While primary prevention through tobacco and alcohol control is essential, the persistently late stage at diagnosis and the concentration of disease burden in high-risk groups argue for complementary secondary prevention strategies that encompass early detection and screening [1]. Recent evidence supports the effectiveness of screening targeted to high-risk populations in reducing OSCC incidence and mortality when embedded within quality-assured pathways for diagnosis and treatment [1]. However, there are no current guidelines for OSCC screening.

Building on this IARC evidence base, this narrative review addresses the lack of clear, practical guidance for developing organized OSCC screening in settings marked by social vulnerability and limited healthcare resources. To help fill this gap, we review the literature and propose a concise theoretical framework that translates established cancer screening principles into operational criteria applicable to OSCC, with a focus on feasibility and equity in low- and middle-income countries (LMICs) as well as socially marginalized subpopulations within high-income settings.

## 2. Methods

An electronic search was conducted in PubMed/MEDLINE and Scopus using the terms “screening”, “cancer”, “oral cancer”, and “principles”, combined through the Boolean operators AND and OR. Articles that referred to screening for cancer, including conceptual foundations, principles of early detection, implementation strategies, and challenges related to screening programs across different cancer types, were considered for inclusion. Because this is a narrative literature review, no date or study design restrictions were applied. Additional manual screening of reference lists from relevant articles was performed to identify further publications of interest. Studies not available in English, conference abstracts, editorials without substantial conceptual contribution, and papers unrelated to screening principles were excluded. The selection process prioritized conceptual, theoretical, and policy-oriented publications that provide insights into principles applicable to cancer screening broadly and to oral cancer in particular. The extracted information was synthesized narratively, emphasizing key themes, recurring concepts, and gaps in the existing literature. This review follows the Scale for the Assessment of Narrative Review Articles (SANRA) principles [4].

## 3. Literature Review

### 3.1. From Wilson & Jungner to Contemporary Screening Principles, Implications for OSCC

Classic screening theory, described by Wilson & Jungner [5], remains foundational but has been refined by decades of experience across cancer sites [6]. Dobrow et al. systematically consolidated 367 unique principles into 12 categories spanning disease/condition, test/intervention, and program/system domains [7]. They highlighted a historical shift in emphasis from disease/test to program/system principles; coordination, quality assurance, data infrastructure, ethics, equity, performance management, and economic evaluation [7]. Smith’s commentary underscores that this is not a wholesale replacement of Wilson & Jungner screening theory, but a refinement that adds a post-implementation guide to sustain and evolve programs in real-world systems [5,6].

For OSCC, which lacks a single universally adopted screening test [8] and operates within health systems with marked heterogeneity. These program-level principles are decisive: without reliable invitation and recall, registry-enabled tracking, quality management, and timely-bound referral, diagnosis, and treatment, the performance of the test itself is insufficient to generate population-level benefit.

### 3.2. The U.S. Preventive Services Task Force (2014): Limited Scope

In 2014, the USPSTF issued a statement for oral cancer screening, concluding that evidence was insufficient to assess the balance of benefits and harms of screening asymptomatic adults in U.S. primary care settings [9]. This recommendation explicitly excluded dental and specialty care settings and was designed for a low-incidence, high-resource healthcare context. Its conclusions were based on heterogeneous accuracy studies, did not evaluate the performance of organized screening programs, and preceded a decade of additional evidence from high-risk regions worldwide. As a result, the applicability of the USPSTF position to current global realities, particularly to LMICs with high prevalence of tobacco, alcohol, smokeless tobacco, and areca-nut exposure, may be limited.

### 3.3. IARC’s Global Evidence

In contrast, the recent IARC international evaluations synthesize evidence from high-incidence regions and conclude that clinical oral examination (COE) can reduce late-stage disease and mortality among high-risk groups when delivered as part of a quality-assured, organized screening pathway [1,10]. These assessments incorporate long-term randomized trials and cohort studies from South Asia and other high-burden settings, providing clearer insights into program effectiveness, determinants of impact (coverage, compliance, follow-up), and implementation challenges [1,10]. Evidence on program performance (number of screened individuals, relative risks) was summarized in the IARC Handbooks of Cancer Prevention, Volume 19: Oral Cancer Prevention [1]. This updated global evidence base supports the need for translating general cancer-screening principles into operational, context-appropriate criteria for organized OSCC screening, particularly in LMICs and settings marked by social vulnerability.

### 3.4. Why OSCC Requires a Targeted, Organized Approach

The global distribution of OSCC reflects inequities in exposure, access, and timeliness of care. In LMICs and underserved subpopulations within high-income settings, diagnostic delays, fragmented referral pathways, and limited access to specialist services contribute to advanced-stage presentation and worse outcomes [1,11,12,13]. Within this context, the IARC perspective emphasizes that organized screening focused on high-risk individuals (e.g., current/former tobacco users, alcohol users, immunosuppressed and socially vulnerable populations) represents a pragmatic strategy to deliver measurable population benefit, but only when screening is embedded within assured pathways for confirmatory diagnosis and timely treatment [1,10].

This aligns with implementation-oriented recommendations that call for pilot programs in high-prevalence settings, either as add-ons to existing public programs (e.g., tobacco cessation or lung-cancer screening, opportunistic screening in medical and dental visits) or as stand-alone pilots focused on high-risk cohorts, deliberately designed to generate data on participation, detection, compliance, resource use, cost, and long-term outcomes [14,15].

### 3.5. Organized Screening Essential and Desirable Criteria Applied to OSCC

An international consensus led by Zhang et al. assembled criteria for an “organized” screening program to replace the simplistic dichotomy of “organized vs. opportunistic” with a graded, implementable framework [16]. Notably, 16 essential criteria received consensus. The strongest agreement for the presence of a protocol/guideline specifying the target population, intervals, tests, referral pathways, management of positives, and a system to identify eligible individuals. Other essentials include invitation systems, call–recall, performance monitoring with reference standards, audit, quality assurance, information systems with linkages (e.g., registries), continued training, and transparent dissemination of performance. Among the desirable criteria, a designated organization/team for implementation/coordination and equity of access are prominent [16].

These criteria are directly translatable to OSCC screening: programs must (1) explicitly define high-risk eligibility; (2) implement invitation and recall; (3) ensure rapid confirmatory diagnosis (including oral medicine/oral pathology assessment and biopsy); (4) track time-to-diagnosis and time-to-treatment; and (5) operate a closed-loop information system to minimize loss to follow-up. Two additional insights from Zhang et al. are highly relevant to OSCC. First, “organized” is not synonymous with “population-based”, the latter is one essential feature. Program organization requires multiple coordinated conditions. Second, the global delivery ecosystem is changing, with shifts in public/private, not-for-profit, and insurer-led programs assuming functions traditionally held by ministries of health (e.g., call–recall, quality assurance, registries) [16]. For OSCC, mixed public–private contexts should be viewed as opportunities to anchor organized screening within existing administrative and information infrastructures, provided equity safeguards are explicit.

### 3.6. The E.A.S.Y. Framework

Operationalizing screening principles requires the use of clear tools and metrics. Wang et al. propose the E.A.S.Y. framework, *Education*, *Assessment*, *Screening*, and *Yield*, as a quality-improvement structure for real-world screening programs. The framework highlights the need for explicit policies and protocols. Quality control throughout the screening pathway, indicator-based monitoring, transparent organizational processes, and systematic risk assessment to focus efforts where benefit is greatest. In addition, operational frameworks should incorporate economic and outcome metrics, including cost per detection, referral and care costs, and cancer outcomes [17].

#### 3.6.1. Education

Education involves targeted awareness efforts, especially among high-risk groups and frontline providers. A toolkit comprises a collection of evidence-based strategies, protocols, educational materials, and practical tools designed to guide health-care providers, community leaders, and policymakers in the effective implementation of initiatives that improve health outcomes and quality of life [18]. Toolkits function as a form of knowledge translation bringing together educational and functional resources to support behavioral change and the adoption of best practices [19]. In the context of cancer, toolkits consolidate guidance on education, early detection, screening, and access to care, tailored to the prevention of specific cancer types. Toolkits for oral cancer prevention have been developed in high-income countries, including the United Kingdom [20] and the United States [21].

#### 3.6.2. Assessment

Assessment involves the incorporation of systematic risk stratification into clinical workflows [17]. Targeting screening programs toward high-risk populations improves the performance of the primary test and enhances the effectiveness of detecting individuals with OSCC [1,10,22].

#### 3.6.3. Screening

Louredo et al. identified substantial heterogeneity in the COE methods employed in head and neck cancer screening programs [8]. Despite the diversity of COE methods applied, studies demonstrate that its sensitivity for detecting OSCC and OPMDs ranges from 50% to 99%, with specificity between 75% and 99% [1,10].

Evidence has been recently validated by IARC on the effectiveness of screening programs and COE for OSCC detection [1,10]. COE is an effective approach for early detection of OSCC in targeted screening programs of high-risk populations, the effectiveness is intrinsically linked to examiner competence, experience and the clinical manifestations of disease. This may be enhanced with training and calibration of examiners prior to implementation in screening [23].

Adjunctive approaches, such as vital staining (toluidine blue), fluorescence visualization, mucosal transillumination, oral cytology, and salivary biomarker assays, may offer incremental diagnostic value but currently require stronger evidence from well-designed randomized trials before routine implementation [1].

#### 3.6.4. Yield

Yield includes monitoring diagnostic timeliness and treatment initiation. Beyond conceptual frameworks, effective screening programs depend on robust information systems. Population-based registries enable invitation and call-recall processes, and linkage between screening, diagnosis, and treatment. These systems require quality monitoring. CanScreen5 illustrates how harmonized indicators and quality assessment of data \support program organization and benchmarking across diverse settings [24]. Evidence shows that programs lacking information systems struggle to achieve adequate coverage, follow-up, and continuity of care, whereas registry-enabled programs demonstrate stronger performance [24].

Vázquez et al. show that multi-component interventions anchored in primary health care, including continuing provider education, fast-track referral protocols, and patient navigation, can improve early diagnosis when adapted to local contexts [25]. These lessons are directly applicable to OSCC, where success depends on coordinated engagement between primary care and dental services, rapid access to oral medicine and oral and maxillofacial pathology expertise, and context-sensitive implementation strategies.

### 3.7. Artificial Intelligence (AI) Integration in Screening Programs

Artificial intelligence might offer advantages for cancer screening. This may include improved diagnostic accuracy, shorter reading times, reduced workload for specialists, and potential gains in efficiency and cost-effectiveness. However, these benefits accrue in individuals who participate in screening, and do not address the persistent challenge of non-attendance [26]. AI is actively being evaluated in cancer screening programs, especially for breast, lung, and colorectal cancers [27,28,29,30,31,32]. For OSCC, AI-assisted image analysis may also enhance the performance of COE [33,34,35].

## 4. Proposal of a Concise Framework for OSCC Organized Screening Program in LMICs

Aa pragmatic program design for OSCC screening in LMICs is proposed (Table 1). The *Education* principle should include a toolkit focused on health care professionals’ education, encompassing primary prevention, focused on health literacy of the general population on OSCC and risk factors control, and early detection.

*Assessment* should prioritize risk-stratified screening, rather than undifferentiated age-based population approaches. While age-band anchoring (e.g., 18–40 every 3 years; 41–69 annually; ≥70 individualized to clinical context) can support baseline consistency. Program benefits will depend on risk-factor weighting, with tobacco users and alcohol users warranting more frequent examination independent of chronological age.

For *Screening*, the COE remains the foundational test. Given geographic distribution and concentration of specialist expertise, dental schools may represent valuable regional hubs for organized OSCC screening in LMICs [36,37]. Dental schools commonly inlude multidisciplinary teams (oral medicine, oral and maxillofacial pathology, and oral surgery) and are closely connected to head and neck surgery, clinical oncology, and radiation oncology, allowing them to operate as integrated referral centers.

A critical consideration in planning and interpreting oral cancer screening programs is the balance between true and false results, particularly in the context of a low-incidence disease. As emphasized in the literature, the accuracy of COE varies widely across settings, with sensitivity ranging from 0.50 to 0.99 and specificity generally above 0.80 [1]. These performance parameters directly influence the rates of false positives and false negatives. False-positive screens may lead to unnecessary psychological distress, additional clinical visits and procedures which carry their own risks and resource implications. Conversely, false-negative results pose an even more serious concern as individuals may be incorrectly reassured that leads to delay in diagnosis with potential disease progression. [15]. The Kerala randomized trial demonstrated the life-saving potential of early detection, but even within this rigorously conducted program, the sensitivity of visual examination remained suboptimal, underscoring the inherent challenge of distinguishing OPMDs and early cancers from benign look-alike lesions [38]. These realities illustrate why continuous training, careful test validation, structured pathways for confirmatory diagnosis and care, and ongoing quality assurance are essential. Feasibility and pilot studies, including infrastructure availability, and quality assurance audits, are essential. Performance indicators (e.g., attendance among the eligible population (%), positivity rate (%), referral further assessment rate (%), further assessment participation rate (%)) may help identify domains needing improvement before scaling up.

Dental schools can reasonably fulfill (or coordinate) many essential criteria: protocol authorship and training, eligibility identification (via networks with public primary care and private providers), invitation/call–recall (by leveraging school IT), quality assurance, registry/indicator management, and rapid diagnostic pathways. Importantly, other institutional nodes may also serve as complementary screening hubs, for example, comprehensive cancer centers, national referral hospitals, or primary care networks with embedded teleconsultation capacity, particularly in settings where university-based dental schools are sparse or regionally concentrated. These entities also have the potential to interface with both public payers and private insurers. Pilot programs might be funded by research groups and local health authorities, but as programs scale, public health systems (and private insurers, in mixed systems) should be accounted for in cost-sharing arrangements. Leveraging dental schools as regional hubs has the potential to expand service capacity and to enable academically teams to publish program outcome data, thereby strengthening transparency, reproducibility, and continuous quality improvement.

For the *Yield* principle, a minimal viable registry should capture eligibility, invitations, attendance, screening findings using standardized lesion descriptors, referrals, oral pathology results, and treatment milestones. Dashboards tracking core indicators such as coverage, positivity rate, compliance with referral, and diagnostic intervals can be developed. Integrated early detection pathways are essential to ensure that screening benefits translate into timely care. In addition, the oral/dental assessment available is an important in the next steps in oncology care if a diagnosis of cancer is made.

To accelerate learning, this perspective calls for implementation research through an OSCC screening pilot program focusing on high-risk cohorts. Designed from inception to collect the data needed for future scale-up and economic evaluation (uptake, compliance, detection, stage shift, resource use, costs, overdiagnosis, sustainability). Combining dental school hubs and/or other eligible tertiary-level institutions, with defined workflows, a screening registry, and fast-track referral pathways, offer a coherent translational route from pilot to program. In parallel, the program may incorporate any advances in diagnosis molecular assessment, and potential genetic testing for well-characterized hereditary risk clusters [39].

## 5. Conclusions

OSCC imposes a substantial burden that justifies targeted, organized screening for high-risk groups when delivered within quality-assured diagnostic and care pathways. Evidence on screening emphasizes test performance, and governance, information systems, coordinated referral pathways, and equity. Continued development and validation of screening principles and screening adjuncts remain essential. By aligning essential and desirable criteria for program organization with practical operational enablers, health systems can design feasible pilot programs to inform ongoing planning. Additionally, the development and validation of screening adjuncts capable of reducing false positives and false negatives, while improving positive predictive value, will further enhance patient care and support safer, more efficient diagnostic pathways and proper treatment, improving prognosis and quality of life.

## Figures and Tables

**Table 1:** Translation of contemporary screening concepts [7,16,17] to a framework for organized OSCC screening.

Contemporary Principles [7,16,17]	Framework for OSCC Organized Screening
Epidemiology of the disease or condition	High incidence and mortality rates of OSCC in LMICs and socially marginalized subpopulations within high-income settings, especially in men. There is a high prevalence of high-risk habits in LMICs population and underserved subpopulations in high-income settings. The most common risk factors are tobacco use, and alcohol consumption.
Natural history of disease or condition	OSCC might be preceded by OPMDs, such as leukoplakia, erythroplakia, and proliferative verrucous leukoplakia. Nonetheless, a substantial proportion of OSCC cases can arise in the absence of identifiable risk factors or clinically recognized precursor lesions and increaseingly in patients without identified risk fators
Target population for screening	Individuals who are tobacco (smoked or smokeless) and/or alcohol consumers, immunosuppressed, and especially over 40 years old, as incidence. Individuals living in social vulnerability settings (LMIC populations and underserved subpopulations in high-income settings) and with low level of health literacy are also at risk.
Screening test performance characteristics	COE is an effective screening test for high-risk individuals. Then, standardized COE protocol and recall schedules tailored to age and risk (18–40 every 3 years; 41–69 annually; ≥70 individualized; annual for tobacco/alcohol users independent of age).
Interpretation of screening test results	Examiners must be periodically trained and calibrated to improve early detection rate. An educational toolkit providing material for primary and secondary prevention of OSCC in screening is a valuable strategy.
Post screening test options	Defined referral pathways for biopsy and specialist assessment are essential. Metrics for time-to-diagnosis and time-to-treatment are necessary indicators. Involving regional hubs (e.g., dental schools, teaching hospitals, and academic clinics) might reduce inequities in access and intervals from a positive-COE to diagnosis.
Screening program infrastructure	Primary health care settings might identify through risk-assessment questionnaires (criteria described in row ~Screening test performance characteristics~) and contact the high-risk population, advise, and promote tobacco and alcohol cessation interventions. Screening invitation should include all individuals enrolled in tobacco and alcohol cessation programs. Information systems to help identify, call-recall, and track incidence and mortality rates of OSCC. Dental schools, teaching hospitals, and academic clinics might play a key role in facilitating access to biopsy and specialist assessment, timely diagnosis, and treatment.
Screening program coordination and integration	The OSCC screening program must be supported by health care systems, from the first stage as a pilot program until full-scale-up to a population-based program. Integrating regional hubs helps with transparency and quality improvement. In addition, there is a need for multisectoral governance (ministries of health, academic centers, and local health authorities) and interoperability with national cancer registries, pathology networks, and fast-track referral systems.
Screening program acceptability and ethics	The program should include uptake statistics (invitation and screening coverage (%)), written informed consent (at first enrollment), ethical audits on equity to access (e.g., qualitative studies analyzing user experience and external audit), communication practices, and acceptability surveys (e.g., how the population understands OSCC, the purpose of screening, and cultural sensitivity). Community engagement in screening activities might improve coverage.
Screening program benefits and harms	Expected benefits include reduction in the detection rate of advanced stage OSCC, and mortality rates. Potential harms may include false positives-COE, false negative results.
Economic evaluation of screening program	Cost analysis, cost-effectiveness, cost-benefit, and budget impact analysis are essential indicators for an OSCC screening program, as they demonstrate the potential impact of the investment prior to scale-up. Funding must be addressed.
Screening program quality and performance management	Use of dashboards for monitoring coverage, positivity, compliance, intervals, and outcomes. Data elements selected to inform scale-up: coverage, compliance, detection, stage shift, cost, overdiagnosis, and sustainability.

Legend: COE, clinical oral examination; LMICs, low- and middle-income countries; OPMD, Oral Potentially Malignant Disorder; OSCC, oral squamous cell carcinoma.

## Data Availability

No new data were created or analyzed in this article.
